# Whey Peptide Alleviates Muscle Atrophy by Strongly Regulating Myocyte Differentiation in Mice

**DOI:** 10.3390/medicina60030433

**Published:** 2024-03-05

**Authors:** Jin A Lee, Mi-Rae Shin, Minju Kim, Hwa-Young Kim, Hwang-Yong Choi, Yoojin Seo, Hakjoo Choi, Seong-Soo Roh

**Affiliations:** 1 Department of Herbology, Daegu Haany University, Deagu 42158, Republic of Korea; tgs02022@naver.com (J.A.L.); with750@naver.com (M.-R.S.); 2 Research Center for Herbal Convergence on Liver Disease, Daegu Haany University, 1, Hanuidae-ro, Gyeongsan-si 38610, Republic of Korea; mj8976@naver.com; 3 Ju Yeong NS Co., Ltd., Seoul 05854, Republic of Koreahychoi@juyeongns.com (H.-Y.C.);

**Keywords:** muscle atrophy, whey peptide, dexamethasone, glucocorticoids, myocyte differentiation

## Abstract

*Background and Objectives*: Muscle atrophy occurs when protein degradation exceeds protein synthesis, resulting in imbalanced protein homeostasis, compromised muscle contraction, and a reduction in muscle mass. The incidence of muscle atrophy is increasingly recognized as a significant worldwide public health problem. The aim of the current study was to evaluate the effect of whey peptide (WP) on muscle atrophy induced by dexamethasone (DEX) in mice. *Materials and Methods:* C57BL/6 mice were divided into six groups, each consisting of nine individuals. WPs were orally administered to C57BL/6 mice for 6 weeks. DEX was administered for 5–6 weeks to induce muscle atrophy (intraperitoneal injection, i.p.). *Results*: Microcomputer tomography (CT) analysis confirmed that WP significantly increased calf muscle volume and surface area in mice with DEX-induced muscle atrophy, as evidenced by tissue staining. Furthermore, it increased the area of muscle fibers and facilitated greater collagen deposition. Moreover, WP significantly decreased the levels of serum biomarkers associated with muscle damage, kidney function, and inflammatory cytokines. WP increased p-mTOR and p-p70S6K levels through the IGF-1/PI3K/Akt pathway, while concurrently decreasing protein catabolism via the FOXO pathway. Furthermore, the expression of proteins associated with myocyte differentiation increased noticeably. *Conclusions*: These results confirm that WP reduces muscle atrophy by regulating muscle protein homeostasis. Additionally, it is believed that it helps to relieve muscle atrophy by regulating the expression of myocyte differentiation factors. Therefore, we propose that WP plays a significant role in preventing and treating muscle wasting by functioning as a supplement to counteract muscle atrophy.

## 1. Introduction

Muscle contains the largest storage of protein in the body and is one of the most important tissues. It plays various roles, including respiration, locomotion, and glucose homeostasis [1]. Muscle atrophy caused by various circumstances such as aging can lead to diseases such as myopathy and muscular dystrophy and various complications such as diabetes, heart failure, and cancer [2]. The incidence of muscle atrophy worldwide is increasing annually, burdening patients’ quality of life; it is thus recognized as a major public health problem [3]. Muscle atrophy occurs when there is an imbalance in protein homeostasis, characterized by excessive protein degradation instead of synthesis. The condition is characterized by decreased muscle mass, muscle contraction, and atrophy of the muscle fiber cross-sectional area (CSA) [4].

Many factors are involved in muscle atrophy. Insulin-like growth factor-1 (IGF-1) increases skeletal muscle protein synthesis through the PI3K/Akt/mTOR pathway [5]. PI3K phosphorylates mTOR and activates Akt, which consequently induces muscle growth and protein synthesis. In contrast, a decrease in Akt activation induces muscle loss by increasing the expression of MuRF-1 and MAFbx, which induce proteolysis through the increase in transcription by FOXO [6,7]. Therefore, maintaining a balance between the PI3K/Akt/mTOR and FOXO pathways is crucial for protein homeostasis in the context of muscle atrophy.

Whey protein, a supplement widely used in the food and pharmaceutical industries, is composed of high levels of β-lactoglobulin, α-lactalbumin, lactose, lactoferrin, and vitamins [8]. Whey protein has been used in various products in the sports nutrition market based on research findings that report that it contains all essential amino acids and helps promote muscle growth [9]. Whey peptide (WP), a hydrolyzed form of whey protein, is known to have stronger cell affinity and specificity than whey protein and also shows a higher absorption rate in the body [10]. In addition, various studies have shown that WP prevents various diseases due to its immunomodulatory and various other effects, such as anti-inflammation, mitigation of oxidative damage, improvements in muscle damage, improvements in motor function, and reductions in body fat [11,12,13,14,15]. Currently, many studies are being conducted on the effects of WPs on muscles [16,17,18,19].

This study aimed to determine the effect of WP, a hydrolyzed form of whey protein, on muscle protein synthesis and degradation. Dexamethasone (DEX), a type of glucocorticoid, was used to induce muscle atrophy in animals. Glucocorticoids are recognized as one of the causal factors contributing to muscle atrophy [20]. Muscle atrophy induction and experiment progressed as follows: WPs were orally administered to C57BL/6 mice for 6 weeks. DEX was administered for 5–6 weeks to induce muscle atrophy (intraperitoneal injection, i.p.). Subsequently, muscle tissue was analyzed to investigate changes in factors affecting muscle protein synthesis and degradation.

## 2. Materials and Methods

### 2.1. Test Material

WP was supplied by Ju Yeong NS Co., Ltd. (Seoul, Republic of Korea). It was manufactured by Tatua Cooperative Dairy Company Limited (Morrinsville, New Zealand) and provided by Megmilk Snow Brand Co., Ltd. (Tokyo, Japan).

The concentrations of WPs for oral administration were 500 mg/kg and 1000 mg/kg, and the concentration of whey protein concentrate for oral administration was 1000 mg/kg. In addition, the concentration of oxymetholone for oral administration was 50 mg/kg.

### 2.2. Reagents

The DEX (Cat. No. D2915) used in this experiment was purchased from Sigma-Aldrich Co. (St. Louis, MO, USA). IGF-1 (SC-518040), PI3K (SC-1637), p-mTOR (SC-293133), mTOR (SC-517464), p-p70S6K (SC-8416), p70S6K (SC-8418), GDF-8 (Myostatin, SC-134345), SIRT1 (SC-15404), MuRF-1 (SC-398608), MAFbx (SC-166806), MyoD (SC-377460), Myogenin (SC-12732), SIX-1 (SC-514441), β-actin (SC-47778), and Histone-1 (SC-8030) were purchased from Santa Cruz Biotechnology (Dallas, TX, USA). p-PI3K (#4228), p-Akt (#9275), Akt (#9272), p-FOXO3a (#9465), and FOXO3a (#12829) were purchased from Cell Signaling Technology, Inc. (Danvers, MA, USA). MYF5 (ab125301) was purchased from Abcam, Inc. (Cambridge, UK). In addition, MYF6 (MRF4, #PA5-97990) was purchased from Thermo Fisher Scientific, Inc. (Waltham, Middlesex County, MA, USA). Secondary antibodies were purchased from GeneTex, Inc. (Irvine, LA, USA). Blotting detection reagents for Western blotting were purchased from ECL Western Cyanagen Srl (Bologna, Italy). Nitrocellulose membranes were purchased from Amersham GE Healthcare (Little. Chalfont, UK).

### 2.3. Experimental Animals

Four-week-old male C57BL/6 mice (Daehan Bio Link Co., Ltd., Chungbuk, Republic of Korea) were purchased and allowed to acclimate in the laboratory environment for a week before conducting the experiments. The animal room conditions were controlled according to the following conventional system: Temperature of 22 ± 2 °C, humidity of 50% ± 5%, and 12 h light/dark cycles. Food (≥18% crude protein, ≥5% crude fat, ≤5% crude fiber, ≤8% crude ash, ≥1% calcium, ≥0.85% phosphorus, ≥0.55% potassium, ≥0.25% sodium, and ≥0.15% magnesium; NIH-41, Zeigler Bros, Inc., Gardners, PA, USA) and water were supplied in sufficient quantities. Animal Treatment and maintenance were performed according to the Guide for the Care and Use of Laboratory Animals of Daegu Haany University (Daegu, Republic of Korea). All protocols for animal experiments were approved by the Daegu Haany University Animal Care and Use Committee, approval number: DHU2022-006.

### 2.4. Muscle Atrophy Induction and Treatment

**Figure 1:**
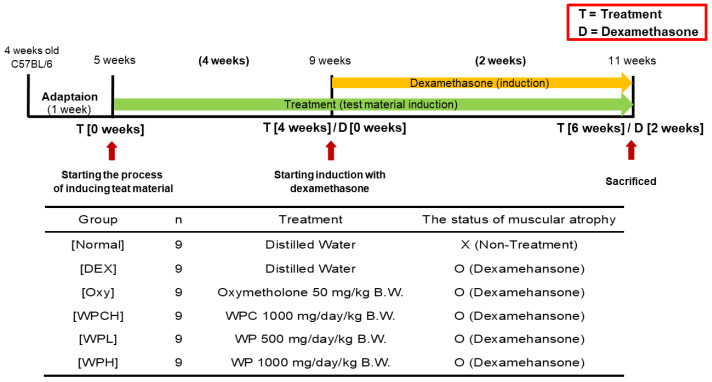
Experimental design.

The experimental groups were divided into six groups of nine individuals, each as follows: Normal (normal group), DEX (DEX-induced muscle atrophy mice treated with distilled water), Oxy (DEX-induced muscle atrophy mice were treated with oxymetholone 50 mg/kg body weight), WPCH (DEX-induced muscle atrophy mice treated with whey protein concentrate at 1000 mg/kg body weight), WPL (DEX-induced muscle atrophy mice were treated with WP at 500 mg/kg body weight), and WPH (DEX-induced muscle atrophy mice treated with WP at 1000 mg/kg body weight). The body weights of all mice were measured once at the same time every day. WPs, at a dosage of 500 and 1000 mg/kg, and whey protein concentrate at a dosage of 1000 mg/kg [21,22] were orally administered daily for 6 weeks. The treatment started 4 weeks prior to DEX administration. In addition, oxymetholone was orally administered simultaneously with WP and whey protein concentrate at 50 mg/kg. DEX was administered subcutaneously once daily at a dosage of 1 mg/kg for 14 days, to induce muscular atrophy [23]. Instead of administering WP, whey protein concentrate, or oxymetholone, mice in both the Normal and DEX groups were given an equal dose of distilled water orally. Simultaneously, the Normal group was subcutaneously injected with saline instead of DEX (Figure 1). The previously selected dose of oxymetholone, 50 mg/kg, was determined based on the efficacy test conducted on mice [24,25]. After completing the experiments, muscle tissues were extracted, and blood was collected from the abdominal aorta. The blood was centrifuged at 1508× *g* for 10 min to separate the serum. The serum and muscle tissues were then stored at −80 °C in a deep freezer.

The body weights of mice were measured once daily. The gastrocnemius (Gast), quadriceps femoris (Quad), tibialis anterior (TA), extensor digitorum longus (EDL), and soleus (Sol) muscles were excised and weighed after the experiments were completed.

### 2.5. Grip Strength Measurement

The mice were placed on a grid, one by one, and a force was applied until just before the mouse fell off the grid. The force applied was measured using a grip strength measuring instrument (Jungdo Bio & Plant Co., Ltd., Seoul, Republic of Korea) right before it fell off the grid. Grip strength was measured thrice over 2 weeks while administering DEX on days 0, 7, and 14.

### 2.6. Histological Analysis

Gast muscle sections (3 μm thickness) were stained with hematoxylin and eosin (H&E) or Sirius Red (Sigma-Aldrich, St Louis, MO, USA). Digital slide scanner (PANNORAMIC 250 Flash III, 3DHISTECH Ltd. H-1141 Budapest, Öv u. 3., Hungary) and then observed using a dedicated view program (Caseviewer, 3DHISTECH Ltd. H-1141 Budapest, Öv u. 3., Hungary).

### 2.7. Blood Analysis

Blood urea nitrogen (BUN), creatinine, aspartate aminotransferase (AST), alanine aminotransferase (ALT), tumor necrosis factor-α (TNF-α), interleukin-6 (IL-6), and interleukin-1β (IL-1β) levels were measured using the separated serum. BUN, AST, and ALT kits were purchased from Asan Pharmaceutical Co., Ltd. (Seoul, Republic of Korea). The creatinine kit was purchased from BioSystems S.A. (Barcelona, Spain). In addition, TNF-α, IL-6, and IL-1β kits were purchased from Koma Biotechnology (Seoul, Republic of Korea).

### 2.8. Micro-Computer Tomography (CT) Measurement

After fixing specimens to a jig for Micro-CT measurement using parafilm, 800 images were obtained using a tube voltage of 90 kV, a current of 88 µA, and a 1.0 aluminum filter (SkyScan1173; Bruker Micro-CT, Kartuizersweg 3B 2550 Kontich, Belgium). The cross sections were reconstructed using NRecon software 2.0 (Bruker Micro-CT). Each of the obtained cross-sectional images was aligned using Dataviewer 1.5, and parameter values were calculated using CtAn software 1.6.

### 2.9. Tissue Western Blotting

Gast muscle was used to evaluate muscle atrophy-related proteins by Western blotting. Many researchers have primarily focused on Gast muscle in models of DEX-induced muscle atrophy. This is because Gast muscle contains both fast-twitch and slow-twitch muscle fibers, making it a mixed fiber-type muscle. Therefore, we also evaluated Gast muscle based on the precedent set by previous researchers. Gast muscle was placed in buffer A (100 mM Tris-HCl (pH 7.4), 5 mM Tris-HCl (pH 7.5), 2 mM MgCl_2_, 15 mM CaCl_2_, 1.5 M sucrose, 0.1 M DTT, and protease inhibitor cocktail). The solution was ground with a tissue grinder (BioSpec Products, Bartlesville, OK, USA) and left on ice for 30 min to obtain the cytoplasm from the muscle tissues. After that, the solution was centrifuged at 13,572× *g* for 2 min after adding a 10% NP-40 solution to separate supernatants containing cytoplasm. The supernatants were rinsed twice in buffer A containing 10% NP-40 to isolate nuclei. Next, 100 µL of buffer C (50 mM HEPES, 0.1 mM EDTA, 50 mM KCl, 0.3 mM NaCl, 1 mM DTT, 0.1 mM PMSF, and 10% glycerol) was added, and the mixture was resuspended and vortexed thrice every 10 min. Finally, supernatants containing nuclei were obtained after centrifugation at 4 °C and 15,800× *g* for 10 min. The obtained supernatants were frozen at −80 °C. Protein samples (12–15 μg) were electrophoresed using 8–14% sodium dodecyl-sulfate polyacrylamide gel electrophoresis (SDS-PAGE) and the acrylamide gel was transferred to a nitrocellulose membrane to measure protein expression in muscle tissues. The prepared membrane was treated with each primary antibody (1:1000), incubated overnight at 4 °C, and then washed five times with PBS-T every 6 min. Next, the secondary antibody (1:3000) that was reactive toward each treated primary antibody was treated at 25 °C for 1.5 h and washed 5 times with PBS-T every 6 min. The membrane was exposed to an enhanced chemiluminescence (ECL) solution, and protein expression was confirmed using Sensi-Q2000 Chemidoc (Lugen Sci Co., Ltd., Seoul, Republic of Korea). The corresponding bands were quantified using ATTO Densitograph Software 2.2.3 (ATTO Corporation, Tokyo, Japan). The quantified values of the proteins identified in the cytoplasmic sample were normalized to β-actin, while the quantified values of the proteins in the nuclear sample were normalized to Histone-1. Each protein level was divided by the protein level of the Normal group and expressed as a relative ratio (fold of Normal).

### 2.10. Statistical Analysis

In vivo values were expressed as means and standard deviations. Post hoc verification was performed with the least significant differences (LSD) test after performing a one-way analysis of variance (ANOVA) test using the SPSS program for Windows version 26 (SPSS Inc., Chicago, IL, USA). Statistical significance for the average difference between each group was verified at *p* < 0.05, *p* < 0.01, and *p* < 0.001.

## 3. Results

### 3.1. Changes in Body Weight and Food Intake

Table 1 shows changes in the body weight and food intake of the muscle atrophy-induced animals. The body weights of the animals in the DEX group were significantly lower than those in the Normal group after experimenting for several weeks (27.23 ± 2.23 g vs. 23.63 ± 1.20 g). However, the body weights of animals in the Oxy, WPCH, WPL, and WPH groups were similar to those observed in the DEX group. The body weights had increased by 8.06 ± 2.01 g on the last day compared with that on the first day of the experiment in the Normal group, whereas the body weights increased by 4.60 ± 0.70 g in the DEX group. All groups that induced muscle atrophy showed an increase of approximately 5 g in body weight gain, but there was no significant difference compared to the DEX group. In addition, the average food intake over the 6-week period for animals in the Normal, DEX, Oxy, WPCH, WPL, and WPH groups was approximately 2.7 g. Therefore, there was no significant difference in food intake in all groups.

### 3.2. Muscle Changes

**Figure 2:**
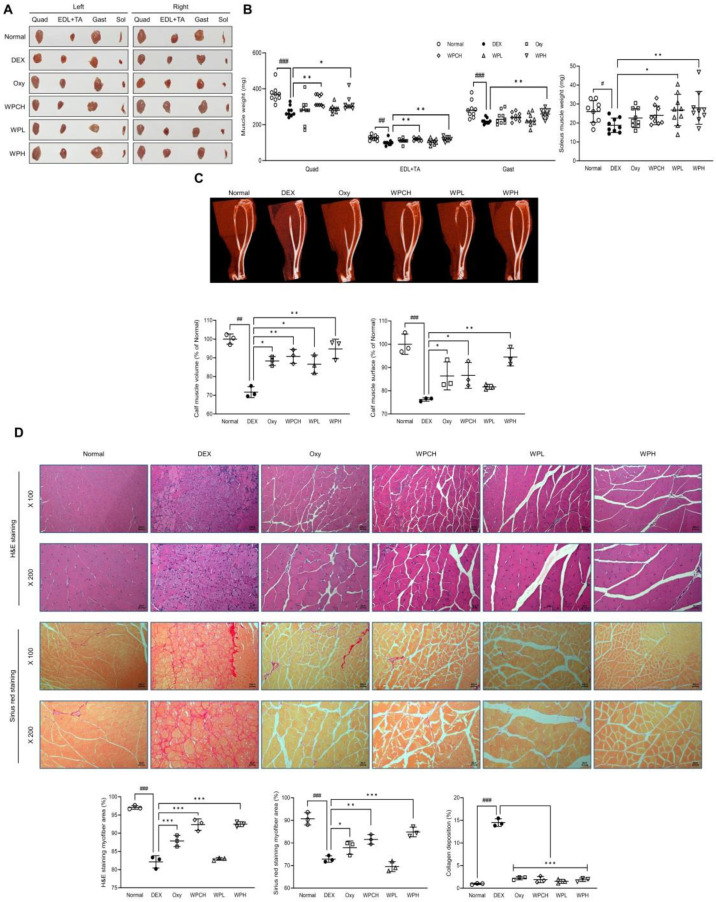
Effects of whey peptide on DEX-induced muscle atrophy: (**A**) a representative gross image of muscles; (**B**) muscle weight; (**C**) micro-computed tomography (CT) of the calf muscle; (**D**) histological examination of muscle through H&E and Sirius Red staining in gastrocnemius muscle (magnification, ×100 and ×200; scale bar, 100 and 50 μm). Normal, normal group; DEX, DEX-induced muscle atrophy mice treated with distilled water; Oxy, DEX-induced muscle atrophy mice treated with oxymetholone at 50 mg/kg body weight; WPCH, DEX-induced muscle atrophy mice treated with whey protein concentrate at 1000 mg/kg body weight; WPL, DEX-induced muscle atrophy mice treated with whey peptide at 500 mg/kg body weight; WPH, DEX-induced muscle atrophy mice treated with whey peptide at 1000 mg/kg body weight. Data are mean ± SD (**B**, n = 9; **C**,**D**, n = 3). Significance: ^#^ *p* < 0.05, ^##^ *p* < 0.01, ^###^ *p* < 0.001 vs. Normal group and * *p* < 0.05, ** *p* < 0.01, *** *p* < 0.001 vs. DEX group. DEX, dexamethasone; H&E, hematoxylin and eosin.

Changes in muscle tissues were investigated after collecting muscle tissues of animals with muscle atrophy induced by DEX. Compared to the Normal group, the weight of muscle tissue was significantly reduced in the DEX group in which muscle atrophy was induced (Quad, *p* < 0.001; EDL + TA, *p* < 0.001; Gast, *p* < 0.001; Sol, *p* < 0.05). Muscle weight significantly increased in both WPH groups (Quad, *p* < 0.05; EDL + TA, *p* < 0.01; Gast, *p* < 0.01; Sol, *p* < 0.01). The weights of Quad (*p* < 0.01) and EDL + TA (*p* < 0.01) were significantly increased in the WPCH group (Figure 2B).

In addition, the volume and surface area of the leg muscles were significantly reduced in the DEX group compared to the Normal group based on the results of the volume and surface area of the leg muscle through the CT scan (volume, *p* < 0.01; surface, *p* < 0.001). In contrast, the volume and surface area of the leg muscles increased significantly in the WPH group compared to the DEX group (volume, *p* < 0.01; surface, *p* < 0.01). Muscle volume and surface area were also significantly increased in the WPCH group compared to the DEX group (volume, *p* < 0.01; surface, *p* < 0.05), and the increase was more prominent in the WPH group than in the WPCH group (Figure 2C).

In addition, changes in muscle fibers were observed by performing H&E and Sirius Red staining. H&E staining revealed that the muscle fibers of the Normal group showed a normal muscle fiber size. However, the size of the muscle fibers in the DEX group was smaller than that in the Normal group. In addition, the size of atrophic and necrotic muscle fibers was observed. In contrast, the muscle fibers of the group administered whey protein concentrate (WPC) and WP showed improved size, unlike the DEX group, which showed atrophic and necrotic muscle fibers. A comparison of the results through quantification showed that the muscle fiber area was significantly reduced in the DEX group than in the Normal group (*p* < 0.001). The area of muscle fibers in the WPH and WPCH groups increased significantly compared with that in the DEX group (*p* < 0.001). Sirius Red staining also showed a significantly lower area of muscle fiber in the DEX group in which muscle atrophy was induced than in the Normal group, similar to the H&E staining result (*p* < 0.001). The area of muscle fibers was significantly higher in the WPH group than in the DEX group, by 16% (*p* < 0.01). In the WPCH group, the muscle fiber area was significantly higher, by 12% (*p* < 0.001). Additionally, collagen deposition was investigated via Sirius Red staining. Consequently, collagen deposition in the DEX group increased significantly compared with that in the Normal group. The collagen deposition over the entire area was confirmed. The collagen deposition was significantly reduced in the groups in which WPC and WP were administered compared to the DEX group (Figure 2D).

### 3.3. Grip Strength Measurement

**Figure 3:**
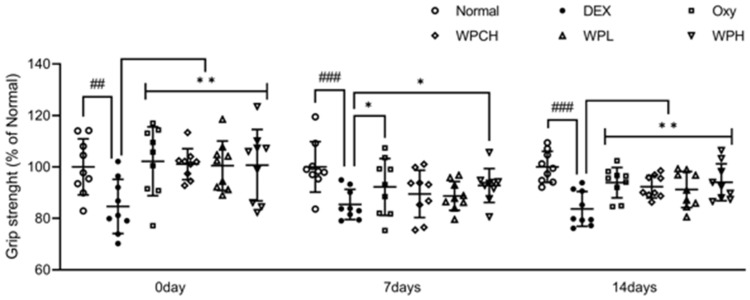
Measurement of grip strength on DEX-induced muscle atrophy. Normal, normal group; DEX, DEX-induced muscle atrophy mice treated with distilled water; Oxy, DEX-induced muscle atrophy mice treated with oxymetholone at 50 mg/kg body weight; WPCH, DEX-induced muscle atrophy mice treated with whey protein concentrate at 1000 mg/kg body weight; WPL, DEX-induced muscle atrophy mice treated with whey peptide at 500 mg/kg body weight; WPH, DEX-induced muscle atrophy mice treated with whey peptide at 1000 mg/kg body weight. Data are mean ± SD (n = 9). Significance: ## *p* < 0.01, ### *p* < 0.001 vs. Normal group and * *p* < 0.05, ** *p* < 0.01 vs. DEX group. DEX, dexamethasone.

Figure 3 shows the results of measuring the grip strength of muscle atrophy-induced mice to investigate the effect of WP on muscle function. After administering Oxy, WPCH, WPL, and WPH treatments for 4 weeks, the DEX group exhibited significantly lower grip strength than the Normal group (100.00 ± 9.73 vs. 84.62 ± 9.44) before muscle atrophy was induced. The grip strength in the Oxy, WPCH, WPL, and WPH groups was significantly higher compared with that in the DEX group, possibly due to the sampling effect. The grip strength 7 days after the induction of muscle atrophy was significantly lower in the DEX group compared to the Normal group (100.00 ± 11.54 vs. 84.67 ± 5.21), similar to the grip strength before muscle atrophy was induced. The grip strength in the WPCH and WPL groups increased by 5% compared with that in the DEX group, while the grip strength in the WPH group increased by 9% compared with that in the DEX group, which was significantly higher. Grip strength 14 days after the induction of muscle atrophy was significantly lower in the DEX group than in the Normal group (100.00 ± 8.09 vs. 83.34 ± 8.55). In the case of the DEX group, the grip strength was lower compared with that at 7 days after muscle atrophy was induced. In contrast, the grip strength in all treatment groups was significantly higher than that of the DEX group. In particular, the decrease in grip strength caused by the induction of muscle atrophy was significantly improved in the WPH group. The grip strength in the WPH group was higher than that in the WPCH group on days 0, 7, and 14.

### 3.4. Analysis of Markers Related to Muscle Damage and Kidney Function

The results of investigating the effects of WP on muscle damage and kidney functions are shown in Table 2. The levels of AST and ALT, which are markers related to muscle damage, were significantly higher in the DEX group than in the Normal group (AST, 12.29 ± 0.63 vs. 29.31 ± 3.97 IU/L; ALT, 4.31 ± 0.36 vs. 7.31 ± 1.36 IU/L). Levels of BUN and creatinine, which are markers related to renal function, were also significantly higher in the DEX group than in the Normal group (BUN, 17.86 ± 0.52 vs. 20.68 ± 0.27 mg/dL; creatinine, 0.19 ± 0.01 vs. 0.62 ± 0.04 mg/dL). In the measurement results of muscle damage-related markers, levels of both AST and ALT were significantly decreased in the WPH group than in the DEX group, and ALT level was significantly decreased in the WPCH group. In addition, BUN and creatinine were significantly decreased in the WPH and WPCH groups than in the DEX group in the measurement results of renal function-related markers. The levels of renal function-related markers in the WPCH and WPH groups were reduced to levels similar to those in the DEX group. However, the level of AST, a marker related to muscle damage, in the WPH and WPCH groups was reduced by 29% and 19%, respectively, compared with that in the DEX group, suggesting that the AST level was reduced more effectively in the WPH group than in the WPCH group.

### 3.5. Analysis of Inflammatory Cytokines in Serum

**Figure 4:**
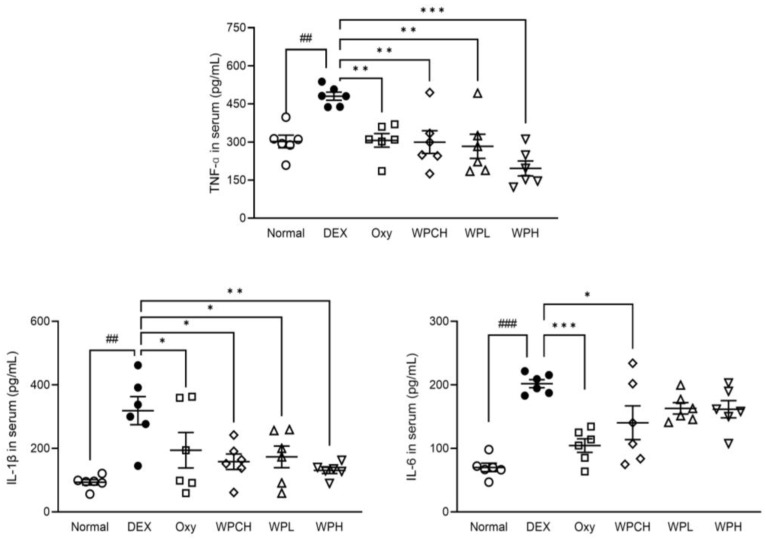
Effects of whey peptide on levels of inflammatory cytokines. Normal, normal group; DEX, DEX-induced muscle atrophy mice treated with distilled water; Oxy, DEX-induced muscle atrophy mice treated with oxymetholone at 50 mg/kg body weight; WPCH, DEX-induced muscle atrophy mice treated with whey protein concentrate at 1000 mg/kg body weight; WPL, DEX-induced muscle atrophy mice treated with whey peptide at 500 mg/kg body weight; WPH, DEX-induced muscle atrophy mice treated with whey peptide at 1000 mg/kg body weight. The shapes on the graphs are displayed differently to make it easier to compare between groups. Data are mean ± SD (n = 6). Significance: ^##^ *p* < 0.01, ^###^ *p* < 0.001 vs. Normal group and * *p* < 0.05, ** *p* < 0.01, *** *p* < 0.001 vs. DEX group. DEX, dexamethasone.

The levels of inflammatory cytokines in the serum are shown in Figure 4. TNF-α, IL-6, and IL-1β levels were significantly higher in the DEX group than in the Normal group. (TNF-α, 301.91 ± 30.15 vs. 480.34 ± 19.45 pg/mL; IL-6, 70.04 ± 8.24 vs. 201.86 ± 7.54 pg/mL; IL-1β, 93.21 ± 10.44 vs. 318.80 ± 53.70 pg/mL). The level of TNF-α was significantly lower in the Oxy, WPCH, WPL, and WPH groups than in the DEX group. The level of TNF-α in the WPCH group, a comparison group, was significantly lower than that in the DEX group by 38%. In particular, the level of TNF-α in the WPH group was significantly lower than that in the DEX group by 59%. Accordingly, the level of TNF-α was reduced by 11% more in the WPH group than in the WPCH group. The level of IL-6 was significantly lower in the Oxy and WPCH groups than in the DEX group. The level of IL-1β was significantly lower in the Oxy, WPCH, WPL, and WPH groups than in the DEX group. In particular, similar to that of TNF-α, the level of IL-1β was 59% lower in the WPH group than in the DEX group. In the WPCH group, the level of IL-1β was reduced by 50% compared with that in the DEX group, indicating that the level of IL-1β was reduced by 9% more in the WPH group than in the WPCH group.

### 3.6. Analysis of Proteins Related to Muscle Synthesis

**Figure 5:**
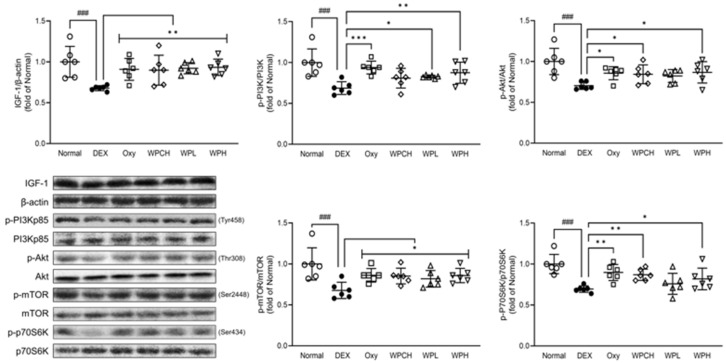
Effects of whey peptide on the protein synthesis pathway in gastrocnemius muscle. Normal, normal group; DEX, DEX-induced muscle atrophy mice treated with distilled water; Oxy, DEX-induced muscle atrophy mice treated with oxymetholone at 50 mg/kg body weight; WPCH, DEX-induced muscle atrophy mice treated with whey protein concentrate at 1000 mg/kg body weight; WPL, DEX-induced muscle atrophy mice treated with whey peptide at 500 mg/kg body weight; WPH, DEX-induced muscle atrophy mice treated with whey peptide at 1000 mg/kg body weight. The shapes on the graphs are displayed differently to make it easier to compare between groups. Data are mean ± SD (n = 6). Significance: ^###^ *p* < 0.001 vs. Normal group and * *p* < 0.05, ** *p* < 0.01, *** *p* < 0.001 vs. DEX group. DEX, dexamethasone.

IGF-1 is known to be involved in skeletal muscle hypertrophy by activating the PI3K/Akt pathway. The PI3K/Akt pathway activated by IGF-1 is involved in cell growth and survival and protein synthesis by inducing phosphorylation of mTOR and its downstream factor p70S6K [26]. As shown in Figure 5, the expression of IGF-1, p-PI3K, p-Akt, p-mTOR, and p-p70S6K was significantly decreased in the DEX group DEX-induced muscle atrophy than in the Normal group. (IGF-1, 0.68 fold; p-PI3K, 0.69 fold; p-Akt, 0.70 fold; p-mTOR, 0.68 fold; p-p70S6K, 0.70 fold). In contrast, IGF-1, p-PI3K, p-Akt, p-mTOR, and p-p70S6K expression increased more in the Oxy, WPCH, WPL, and WPH groups than in the DEX group. In particular, the expression of IGF-1 was significantly increased in the Oxy, WPCH, WPL, and WPH groups, reaching the same level as the Normal group. The expression of all proteins related to muscle synthesis in the WPH group was significantly increased compared to that in the DEX group.

### 3.7. Analysis of Proteins Related to Myolysis

**Figure 6:**
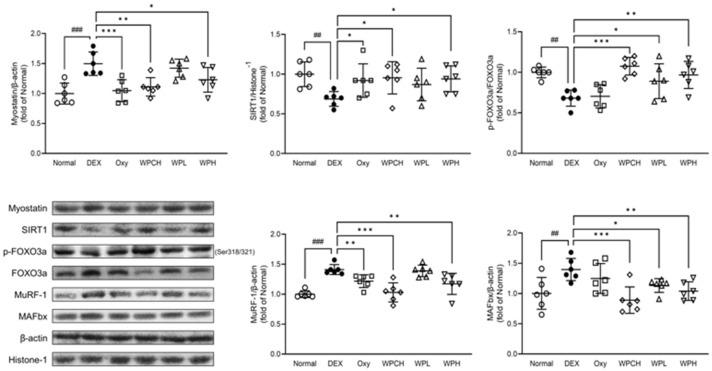
Effects of whey peptide on the protein catabolic pathway in gastrocnemius muscle. Normal, normal group; DEX, DEX-induced muscle atrophy mice treated with distilled water; Oxy, DEX-induced muscle atrophy mice treated with oxymetholone at 50 mg/kg body weight; WPCH, DEX-induced muscle atrophy mice treated with whey protein concentrate at 1000 mg/kg body weight; WPL, DEX-induced muscle atrophy mice treated with whey peptide at 500 mg/kg body weight; WPH, DEX-induced muscle atrophy mice treated with whey peptide at 1000 mg/kg body weight. The shapes on the graphs are displayed differently to make it easier to compare between groups. Data are mean ± SD (n = 6). Significance: ^##^ *p* < 0.01, ^###^ *p* < 0.001 vs. Normal group and * *p* < 0.05, ** *p* < 0.01, *** *p* < 0.001 vs. DEX group. DEX, dexamethasone.

In addition, myostatin and Akt signaling induce protein degradation through muscle loss-related genes such as MuRF-1 and MAFbx through the FOXO pathway [27,28,29]. As shown in Figure 6, the expression of myostatin in the DEX group, in which DEX induced muscle atrophy, increased significantly more than in the Normal group (myostatin, 1.50 fold). However, the expression of SIRT1 and p-FOXO3a was significantly decreased (SIRT1, 0.69 fold; p-FOXO3a, 0.68 fold). In contrast, the expression of these proteins was regulated in the Oxy, WPCH, WPL, and WPH groups than in the DEX group. In particular, protein expression was significantly regulated in the WPH and WPCH groups. The expression of MuRF-1 and MAFbx increased significantly in the DEX group than in the Normal group (MuRF-1, 1.41 fold; MAFbx, 1.40 fold), and the expressions of MuRF-1 and MAFbx were significantly more decreased in the WPCH and WPH groups than in the DEX group. In the WPH and WPCH groups, the expression of SIRT1 and p-FOXO3a related to muscle degradation was significantly regulated, and the expression of its downstream factors, MuRF-1 and MAFbx, was also significantly decreased.

### 3.8. Expression of Proteins Related to Myocyte Differentiation

**Figure 7:**
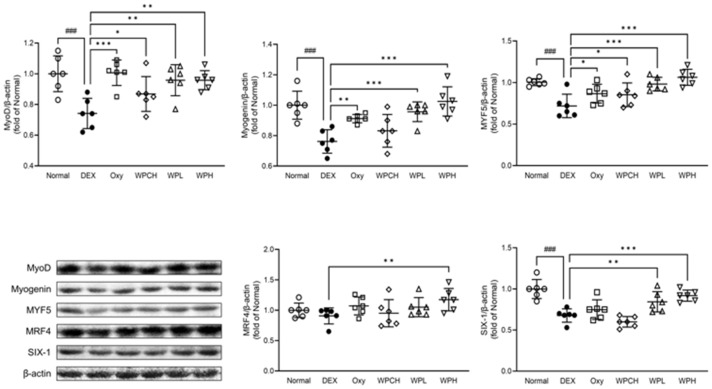
Effects of whey peptide on the myocyte differentiation pathway in gastrocnemius muscle. Normal, normal group; DEX, DEX-induced muscle atrophy mice treated with distilled water; Oxy, DEX-induced muscle atrophy mice treated with oxymetholone at 50 mg/kg body weight; WPCH, DEX-induced muscle atrophy mice treated with whey protein concentrate at 1000 mg/kg body weight; WPL, DEX-induced muscle atrophy mice treated with whey peptide at 500 mg/kg body weight; WPH, DEX-induced muscle atrophy mice treated with whey peptide at 1000 mg/kg body weight. The shapes on the graphs are displayed differently to make it easier to compare between groups. Data are mean ± SD (n = 6). Significance: ^###^ *p* < 0.001 vs. Normal group and * *p* < 0.05, ** *p* < 0.01, *** *p* < 0.001 vs. DEX group. DEX, dexamethasone.

MyoD, myogenin, MYF5, MRF4, and SIX-1, members of the muscle regulatory factor gene family, are known to regulate myocyte differentiation during skeletal muscle development [30,31]. As shown in Figure 7, the expression of MRF4 decreased in the DEX group than in the Normal group (MRF4, 0.91 fold). In addition, the expression of MyoD, myogenin, MYF5, and SIX-1 was significantly decreased (MyoD, 0.74 fold; myogenin, 0.76 fold; MYF5, 0.72 fold; SIX-1, 0.68 fold). In contrast, the expression of all these proteins increased in the Oxy, WPCH, WPL, and WPH groups than in the DEX group. In particular, while the expression of MyoD and MYF5 in the case of the WPCH group, a comparison group, increased significantly, the expression of all myocyte differentiation proteins in the WPL and WPH groups that received the WP significantly increased to the expression level in the Normal group.

## 4. Discussion

Muscle is an important tissue that accounts for approximately 40% of the total body weight and 50% of the total protein. Muscle atrophy is a significant social problem due to a lack of muscle, causing various complications, such as chronic kidney disease, chronic lung disease, diabetes, and obesity [32]. This study aimed to determine the effect of WP, a hydrolyzed form of whey protein, on muscle protein synthesis and degradation. Oxymetholone was used as a comparative drug for the efficacy of WP. Oxymetholone is a drug known to promote muscle growth and has been predominantly used as a comparison drug in muscle atrophy research papers [24,33]. To confirm the efficacy of WP, we orally administered WP to C57BL/6 mice for 6 weeks. DEX was administered for 5–6 weeks to induce muscle atrophy. Subsequently, muscle tissue was analyzed to investigate changes in factors affecting muscle protein synthesis and degradation.

As a result of confirming the changes that WP has on muscle in muscle atrophy, the weight of the Quad, EDL + TA, Gast, and Sol muscles was significantly increased in the WPH group than in the DEX group. Muscle volume and surface area were also increased in the WPL group than in the DEX group based on the results of micro-CT analysis results. In addition, muscle volume and surface area were significantly increased in the WPH group (Figure 2A–C). A recent study utilized immunohistochemical staining to observe an increase in the CSA of both Myosin Heavy Chain I (MyHC I) slow-twitch fibers and Myosin Heavy Chain IIb (MyHC IIb) fast-twitch fibers [14]. After administering WP, the analysis involving H&E and Sirius Red staining revealed similar size enhancements in both the unfilled muscle fibers and small muscle fibers compared with those in the DEX group. Collagen deposition was significantly reduced compared with that in the DEX group (Figure 2D). Increased consumption of WP is known to improve muscle wasting. Our study also showed a positive effect on muscle wasting, which is consistent with the results of previous studies [14].

The levels of AST and ALT, known as markers of muscle damage, rise after muscle damage. However, AST and ALT are highly concentrated in muscle cells. Recent studies have reported that AST and ALT are released into the bloodstream when the muscle is damaged or necrotic without evidence of muscle damage. The levels of AST and ALT, which are involved in amino acid metabolism, increase in muscle-related diseases such as muscular dystrophy and muscle atrophy, resulting in hypertransaminasemia [34,35]. Therefore, the levels of AST and ALT in serum were determined in this study. In addition, the level of BUN and creatinine, a biomarker for kidney disease known to be a representative complication of muscle atrophy, was confirmed. WP reduced AST and ALT levels, which were increased due to muscle atrophy. In particular, their levels were significantly reduced in the WPH group. In addition, WP also significantly reduced the levels of BUN and creatinine, which were increased by muscle atrophy (Table 2).

Changes in the inflammatory cytokines caused by muscle atrophy were investigated through hematological analysis. Inflammatory cytokines such as TNF-α, IL-6, and IL-1β induce muscle wasting by reducing the synthesis of muscle proteins and promoting their degradation [36]. Lang et al. reported that injection of TNF-α induces muscle atrophy. In addition, inflammatory cytokines such as IL-6 and IL-1β exert the same effect through the same mechanism as TNF-α [37]. As previously shown, in this study, inflammatory cytokines were significantly increased in the DEX group in which DEX induced muscle atrophy. WP decreased the inflammatory cytokines that were increased due to muscle atrophy (Figure 4).

To confirm the effect of WP on muscle atrophy, we investigated the expression of muscle protein synthesis and degradation factors and myocyte differentiation factors in Gast muscle through Western blotting. IGF-1 is a major growth factor that regulates both the protein synthesis pathway PI3K/Akt/mTOR and the protein degradation pathway FOXO in muscle. Activation of the PI3K/Akt pathway by IGF-1 phosphorylates mTOR and increases muscle protein synthesis through phosphorylation and activation of p70S6K [5,38]. Huang et al. reported that activating the IGF-1/PI3K/Akt pathway promotes protein synthesis in muscle, suggesting that it is suppressed when this pathway is inhibited [39]. In addition, activation of the PI3K/Akt pathway by IGF-1 inhibits FOXO transcription and regulates protein degradation through mutual regulation [5]. FOXO is a transcription factor involved in metabolism and stress response in the body. Among the FOXO family members, FOXO3 is mainly involved in skeletal muscle atrophy when activated. Additionally, FOXO3 is involved in muscle loss by regulating MuRF-1 and MAFbx [40]. This study showed that the expression of p-mTOR and p-p70S6K, caused by an inhibition of the IGF-1/PI3K/Akt pathway, as previously demonstrated, was decreased in the DEX group with muscle atrophy than in the Normal group. In addition, the expression of the muscle protein degrading factors MuRF-1 and MAFbx was increased through the FOXO pathway.

By regulating the expression of these proteins, WP induced an increase in muscle protein synthesis and suppressed protein degradation. In particular, the expression of all proteins investigated in the IGF-1/PI3K/Akt and FOXO pathways were significantly regulated in the WPH group (Figure 5 and Figure 6). These results suggest that WP alleviates muscle loss by regulating the IGF-1/PI3K/Akt and FOXO pathways.

The expression of myocyte differentiation factors was further investigated in this study. MyoD, myogenin, MYF5, MRF4, and SIX-1, known as muscle regulatory factor gene family members, regulate myocyte differentiation during skeletal muscle development [30,31]. When muscle fibers are damaged, myocyte differentiation factors are activated and regulate muscle loss. These myocyte differentiation factors are major factors that contribute to repairing damaged muscle fibers or forming new muscle fibers [41,42]. In this study, WP significantly increased the expression of myocyte differentiation factors, which were reduced by muscle atrophy. These results suggest that WP helps to alleviate muscle loss by further increasing the expression of myocyte differentiation factors (Figure 7).

## 5. Conclusions

In the present study, WP controlled the downregulation of the FOXO pathway, a muscle protein degradation pathway, and the upregulation of the IGF-1/PI3K/Akt pathway, a muscle protein synthesis pathway. The results are similar to those of the comparative group using whey protein concentrate. Additionally, WP significantly regulated the expression of myocyte differentiation factors and had a superior effect than whey protein concentrate. Overall, this study demonstrated that WP has the ability to prevent and decrease muscle atrophy by influencing the IGF-1/PI3K/Akt and FOXO pathways, and also has a role in promoting muscle growth by regulating the expression of myocyte differentiation factors. 

## Figures and Tables

**Table 1:** Body weight change and food intake.

	Body Weight (g)			Food Intake (g)
	Initial	Final	Gain	
Normal	19.17 ± 0.82	27.23 ± 2.23	8.06 ± 2.01	2.75 ± 0.39
DEX	19.03 ± 0.86	23.63 ± 1.20 ^###^	4.60 ± 0.70 ^###^	2.74 ± 0.60
Oxy	19.26 ± 0.91	24.63 ± 1.52	5.37 ± 1.46	2.79 ± 0.32
WPCH	19.11 ± 0.78	23.98 ± 1.80	4.87 ± 1.57	2.84 ± 0.47
WPL	19.30 ± 0.49	23.75 ± 0.95	4.45 ± 1.15	2.66 ± 0.51
WPH	19.33 ± 0.57	24.20 ± 1.71	4.87 ± 1.86	2.88 ± 0.55

Normal, normal group; DEX, DEX-induced muscle atrophy mice treated with distilled water; Oxy, DEX-induced muscle atrophy mice treated with oxymetholone at 50 mg/kg body weight; WPCH, DEX-induced muscle atrophy mice treated with whey protein concentrate at 1000 mg/kg body weight; WPL, DEX-induced muscle atrophy mice treated with whey peptide at 500 mg/kg body weight; WPH, DEX-induced muscle atrophy mice treated with whey peptide at 1000 mg/kg body weight. Data are mean ± SD (n = 9). Significance: ^###^ *p* < 0.001 versus the Normal group. DEX, dexamethasone.

**Table 2:** Effects of whey peptide on levels of muscle damage and kidney function.

	Muscle Damage (IU/L)		Kidney Function (mg/dL)	
	AST	ALT	BUN	Creatinine
Normal	12.29 ± 0.63	4.31 ± 0.36	17.86 ± 0.52	0.19 ± 0.01
DEX	29.31 ± 3.97 ^###^	7.31 ± 1.36 ^#^	20.68 ± 0.27 ^###^	0.62 ± 0.04 ^###^
Oxy	28.83 ± 2.40	6.45 ± 0.54	19.08 ± 0.36 *	0.58 ± 0.04
WPCH	23.62 ± 3.25	5.08 ± 0.40 *	17.25 ± 0.37 ***	0.52 ± 0.02 *
WPL	21.63 ± 2.09	5.29 ± 0.96	15.16 ± 0.73 ***	0.52 ± 0.02 *
WPH	20.70 ± 3.08 *	4.96 ± 0.48 *	17.64 ± 0.10 ***	0.51 ± 0.03 *

Normal, normal group; DEX, DEX-induced muscle atrophy mice treated with distilled water; Oxy, DEX-induced muscle atrophy mice treated with oxymetholone at 50 mg/kg body weight; WPCH, DEX-induced muscle atrophy mice treated with whey protein concentrate at 1000 mg/kg body weight; WPL, DEX-induced muscle atrophy mice treated with whey peptide at 500 mg/kg body weight; WPH, DEX-induced muscle atrophy mice treated with whey peptide at 1000 mg/kg body weight. Data are mean ± SD (n = 6). Significance: ^#^ *p* < 0.05, ^###^ *p* < 0.001 vs. Normal group and * *p* < 0.05, *** *p* < 0.001 vs. DEX group. DEX, dexamethasone.

## Data Availability

The data used and/or analyzed during the current study are available from the corresponding author upon reasonable request.
